# A Comprehensive Review of the Diagnostic Landscape of Endometriosis: Assessing Tools, Uncovering Strengths, and Acknowledging Limitations

**DOI:** 10.7759/cureus.56978

**Published:** 2024-03-26

**Authors:** Ketki S Dantkale, Manjusha Agrawal

**Affiliations:** 1 Obstetrics and Gynecology, Jawaharlal Nehru Medical College, Datta Meghe Institute of Higher Education and Research, Wardha, IND

**Keywords:** multidisciplinary collaboration, laparoscopy, imaging, biomarkers, diagnosis, endometriosis

## Abstract

Endometriosis is a prevalent yet often underdiagnosed condition characterized by the presence of endometrial-like tissue outside the uterus, leading to significant morbidity and impaired quality of life. A timely and accurate diagnosis of endometriosis is essential for effective management and improved patient outcomes. This review provides a comprehensive overview of the current diagnostic landscape of endometriosis, including clinical evaluation, imaging modalities, biomarkers, and laparoscopy. The strengths and limitations of each diagnostic approach are critically evaluated, alongside challenges such as delayed diagnosis and misinterpretation of findings. The review emphasizes the importance of multidisciplinary collaboration, standardized diagnostic protocols, and ongoing research to enhance diagnostic accuracy and facilitate early intervention. By addressing these challenges and leveraging emerging technologies, healthcare professionals can improve the diagnosis and management of endometriosis, ultimately enhancing the well-being of affected individuals.

## Introduction and background

Endometriosis is a chronic, often painful condition characterized by the growth of endometrial-like tissue outside the uterus, commonly affecting the pelvic organs such as the ovaries, fallopian tubes, and peritoneum [1]. This ectopic tissue responds to hormonal fluctuations during the menstrual cycle, leading to inflammation, scarring, and the formation of adhesions. Endometriosis affects approximately 10% of reproductive-aged individuals and is a leading cause of infertility and debilitating pelvic pain [2].

A timely and accurate diagnosis of endometriosis is paramount due to its significant impact on the physical, emotional, and social well-being of affected individuals. Delayed diagnosis often results in prolonged suffering, impaired quality of life, and increased healthcare costs. Moreover, early identification of endometriosis allows for the prompt initiation of appropriate management strategies, including pain relief, fertility preservation, and disease monitoring [3].

This review aims to comprehensively evaluate the diagnostic landscape of endometriosis, assessing the strengths and limitations of existing tools and techniques. This review seeks to inform clinicians, researchers, and policymakers about the challenges and opportunities in improving diagnostic accuracy and patient care by synthesizing current knowledge and emerging trends in endometriosis diagnosis.

## Review

Current diagnostic tools

Clinical History and Physical Examination

The clinical history and physical examination play a pivotal role in diagnosing endometriosis. Patients with endometriosis typically exhibit symptoms such as heavy menstrual bleeding (menorrhagia), painful menstruation (dysmenorrhea), irregular uterine bleeding (metrorrhagia), persistent pelvic discomfort, and pain during sexual intercourse (dyspareunia). Furthermore, a history of multiple pregnancies or previous uterine surgeries may be noted, with infertility occasionally linked to adenomyosis, particularly as more women postpone childbearing [2,4]. During the physical examination, indicators suggest endometriosis encompasses an immobile retroverted uterus, palpable nodules on the uterosacral ligaments, and a cul-de-sac exhibiting narrowing of the posterior fornix. The presence of an enlarged, tender, and "boggy" uterus often points towards adenomyosis, whereas severe endometriosis is frequently characterized by a fixed, tender uterus with discernible nodules in specific regions [5]. Variations in physical examination findings can be significant, contingent upon the location of endometriotic lesions. For instance, speculum examination may only reveal lesions in a subset of patients, while indicators such as profound dyspareunia and nodules in the pouch of Douglas merit careful consideration [6].

Diagnostic Imaging

Imaging techniques play a pivotal role in the diagnosis of endometriosis. Transvaginal ultrasound (TVS) and magnetic resonance imaging (MRI) stand out as the primary imaging modalities utilized for preoperative assessment and precise identification of endometriosis lesions [6,7]. These techniques are indispensable for discerning various types of endometriotic lesions, encompassing superficial endometriosis, deep endometriosis, and ovarian endometriosis, each demanding tailored imaging approaches for accurate diagnosis [7,8]. Moreover, advanced imaging methodologies such as multidetector computed tomography enema and computed tomography colonography have been investigated to detect bowel endometriosis, offering comprehensive visualization of the bowel wall and aiding in the differentiation from other conditions such as cancer or inflammatory diseases [7]. Although TVS typically serves as the frontline imaging modality owing to its accessibility and cost-effectiveness, MRI assumes particular significance in diagnosing deep infiltrating endometriosis (DIE), notably in regions like neural endometriosis, where ultrasound depiction may be insufficient [8,9].

Biomarkers and Laboratory Tests

Biomarkers serve as vital components in the diagnosis of endometriosis, providing valuable insights into the presence and severity of the disease. These biomolecules encompass diverse substances, including proteins, genes, lipids, RNA, DNA, enzymes, and hormones, reflecting endometriosis's physiological state or condition [10]. While certain individual biomarkers, like CA-125, have been extensively investigated, their diagnostic accuracy may be limited when utilized in isolation. Research indicates that combining multiple biomarkers can enhance diagnostic precision, with certain studies achieving an area under the curve (AUC) ranging from 0.71 to 0.81 for discriminating endometriosis from control subjects [11,12]. Despite sustained research endeavors to identify reliable biomarkers for endometriosis detection, the quest for a singular clinically dependable biomarker still needs to be discovered. The field actively explores emerging technologies such as "omics" approaches, molecular imaging techniques, and microRNAs to bolster diagnostic capabilities [13]. The pursuit of specific diagnostic biomarkers continues to advance, focusing on innovative molecular biology methodologies and diverse monitoring modalities to refine the detection and management of endometriosis.

Laparoscopy: Gold Standard for Diagnosis

Laparoscopy is the gold standard for diagnosing endometriosis, enabling direct visualization of the disease. This minimally invasive surgical technique involves the insertion of a fiber optic camera into the patient's pelvis, facilitating the inspection of internal structures, identification of abnormal tissue areas, and biopsy collection to confirm endometriosis through microscopic examination by a pathologist [14]. While other diagnostic modalities such as imaging studies (MRI, CT, and ultrasound) and medical history may suggest the presence of endometriosis, laparoscopy remains the sole definitive method for accurate diagnosis [14]. Studies have demonstrated that laparoscopic visualization exhibits high sensitivity (90.1%) and moderate specificity (40.0%) compared to histopathology, the gold standard for diagnosis, underscoring its efficacy in detecting endometriotic lesions [15]. Despite its invasive nature, laparoscopy confirms the diagnosis and enables concurrent treatment by surgically excising diseased areas, rendering it an integral component of comprehensive care for patients suspected of endometriosis [14].

Assessing diagnostic accuracy

Sensitivity and Specificity of Diagnostic Tools

The sensitivity and specificity of diagnostic tools for identifying endometriosis are critical for an accurate diagnosis. Different symptoms and physical examination findings exhibit varying levels of sensitivity and specificity in diagnosing endometriosis. Pain exacerbations during menstruation and infertility demonstrate a sensitivity of 20.37% and a specificity of 97.87%. In comparison, symptoms such as intensified menstrual pain and irregular periods present a sensitivity of 75.93% and a specificity of 51.06% [16]. These findings underscore the importance of considering a combination of symptoms and examination results to enhance diagnostic precision. Machine learning algorithms (MLA) have emerged as a novel screening approach for endometriosis, showing promising sensitivity and specificity values ranging from 0.82 to 1 in diagnosing the condition [17]. Furthermore, self-report symptom-based prediction models have exhibited high sensitivity (75%) and specificity (69%) in predicting endometriosis, with the most effective model achieving an AUC of 0.94 [4]. Integrating a combination of symptoms, physical examination findings, and innovative approaches such as MLA and self-report tools can enhance the sensitivity and specificity of diagnostic tools for endometriosis. This integrated approach can facilitate more precise and timely condition identification [16-18].

Challenges in Diagnosis: Delay and Misdiagnosis

Diagnosing endometriosis presents multifaceted challenges, resulting in delays and misdiagnoses. A significant obstacle lies in the diverse manifestations of the disease, rendering small lesions challenging to detect without specific diagnostic tools [19]. Moreover, the nonspecific nature of endometriosis symptoms, such as pelvic pain, heavy menstrual bleeding, and dyspareunia, overlaps with those of other gynecological and gastrointestinal conditions, further complicating the diagnostic journey [20]. The absence of reliable screening tools exacerbates the challenge, as conventional imaging techniques like ultrasound and MRI may not suffice for effective endometriosis detection [20]. Furthermore, the normalization of menstrual pain and a general lack of awareness or education about female health contribute to symptom dismissal or underestimation, leading to delayed or missed diagnoses [20]. This delay in diagnosis, averaging between 7 and 11 years, significantly impacts women's mental health, quality of life, and overall well-being [19]. Additionally, inflammation surrounding abnormal endometrial tissue can complicate the biopsy process, potentially obscuring the microscopic structure necessary for an accurate diagnosis [21]. The complexity of endometriosis symptoms, coupled with the absence of specific diagnostic tools and the normalization of menstrual pain, pose substantial challenges in diagnosing the condition, often resulting in delays and misdiagnoses that profoundly affect women's health and quality of life [19-21].

Patient Perspectives on Diagnostic Experiences

Patient perspectives on diagnostic experiences are crucial for comprehending the intricacies of the diagnostic process. Studies have delved into the interactions and communication dynamics between healthcare providers and patients during diagnostic imaging investigations, shedding light on the importance of patient-centered care and effective communication throughout the diagnostic journey [22]. Patient experiences within diagnostic pathways, such as those observed in lung cancer diagnosis, reveal heightened levels of anxiety associated with fast-track programs, highlighting the necessity for support, information dissemination, and the involvement of relatives to navigate through the diagnostic process effectively [23]. Moreover, research has assessed the impact of various strategies for communicating diagnostic uncertainty on patient perceptions of physician competence. Effectively communicating diagnostic uncertainty can foster patient engagement in the diagnostic process and mitigate delays in seeking appropriate care, thus underscoring the significance of clear and empathetic communication between physicians and patients, particularly during uncertain diagnostic scenarios [24].

Strengths of existing diagnostic approaches

Advantages of Laparoscopy

**Figure 1:**
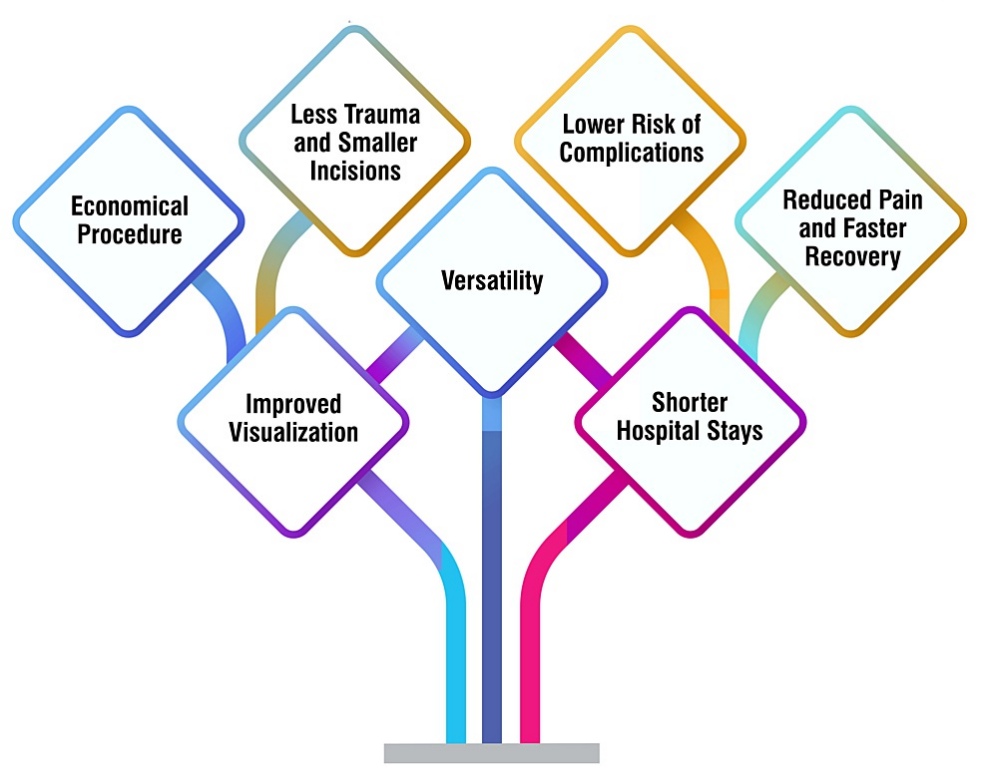
Advantages of laparoscopy Image Credit: Corresponding Author

Laparoscopic surgery offers several advantages over traditional open surgery, making it a preferred choice for many patients and surgeons. Firstly, laparoscopic procedures involve smaller incisions, resulting in less trauma to the body and minimal scarring [25,26]. These smaller incisions also contribute to a lower risk of complications such as infection, blood loss, and swelling, as laparoscopic tools enable precise and complex procedures with reduced trauma to healthy tissues [25]. Additionally, the minimally invasive nature of laparoscopic surgery leads to reduced postoperative pain and faster recovery times compared to traditional open surgery [25]. Furthermore, patients undergoing laparoscopic surgery often experience shorter hospital stays due to the minimally invasive nature of the procedure and the faster healing process [27]. Moreover, laparoscopy is a versatile procedure used for diagnostic and surgical interventions for various conditions, such as endometriosis, fibroids, ovarian cysts, hysterectomy, and more [27]. Another significant advantage of laparoscopy is improved visualization, which provides surgeons with in-depth and realistic insight into body organs and allows for precise and accurate procedures [28]. Additionally, laparoscopic surgery is considered an economical procedure, with benefits including minimum side effects, less internal scarring, and a higher success rate compared to traditional open surgery methods [28]. Overall, these advantages highlight the significant benefits of laparoscopic surgery for patients and healthcare providers alike. The advantages of laparoscopy are shown in Figure 1.

Role of Imaging in Screening and Preoperative Assessment

Imaging is pivotal in screening and preoperative assessment across various medical conditions. In endometriosis, imaging techniques such as CT scans have proven beneficial in preoperative planning for patients with isolated nasal obstruction and septal deviation. A retrospective study underscored the significant contribution of CT imaging in modifying the initial surgical plan based on physical examination findings in most cases, highlighting the importance of imaging in surgical decision-making for such conditions [29]. Similarly, in the assessment and surgical treatment of breast cancer, preoperative magnetic MRI has been instrumental in detecting additional diseases, determining disease extent, and guiding surgical decisions. The utilization of preoperative MRI has been correlated with enhanced detection rates and a more comprehensive understanding of disease extent, thereby facilitating treatment planning and decision-making for patients with breast cancer [30].

Emerging Biomarkers and Their Potential

Emerging biomarkers for endometriosis, including CA-125, CA-199, urocortin, and IL-6, hold promise for detecting the condition [13]. However, despite their potential, these biomarkers still need to meet the criteria for diagnostic biomarkers due to various limitations in their utility and accuracy [13]. Additionally, circulating endometrial cells have been identified as having significant potential for developing an early, non-invasive diagnostic assay for endometriosis [13]. The integration of these promising biomarkers with emerging molecular diagnostic technologies has the potential to unveil new biomarkers for endometriosis in peripheral blood, uterine materials, or urine [13]. While individual biomarkers like CA-125 have undergone extensive study, a panel of multiple markers will likely offer greater accuracy than any single biomarker in diagnosing endometriosis [13]. Further research and validation are imperative to establish clinically reliable, non-invasive tests for endometriosis detection and to enhance patient outcomes [31].

Limitations and areas for improvement

Invasiveness and Risks Associated With Laparoscopy

Laparoscopy is a minimally invasive surgical procedure widely used to diagnose and treat endometriosis. Although generally safe and effective, laparoscopy carries potential risks, including internal bleeding, hernia formation at incision sites, infection, and inadvertent damage to blood vessels or other organs such as the bladder or bowels. Patients may experience post-surgical pain, swelling, or redness and should promptly seek medical attention if they develop a fever or severe symptoms. While most individuals can return home shortly after the procedure, some may necessitate a hospital stay, depending on the complexity of the surgery [32]. Compared to traditional open surgeries like laparotomy, laparoscopy is less invasive. It offers superior visualization of endometriosis lesions, typically leading to shorter hospital stays and quicker recovery times. However, despite its advantages, laparoscopy primarily targets visible lesions, which may not address all aspects of endometriosis-related pain. Additionally, complications such as symptom recurrence, scarring, injuries to adjacent organs like the bladder or bowel, and the requirement for multiple surgeries may arise in some cases. The efficacy of laparoscopic surgery in alleviating symptoms varies among patients, with some individuals experiencing persistent pain even after lesion removal [32,33].

Challenges in Interpreting Imaging Findings

Interpreting imaging findings for endometriosis presents unique challenges that necessitate expertise and experience. Pelvic MRI for endometriosis, in particular, poses a diagnostic challenge requiring a specific skill set and experience due to the intricate nature of the disease presentation. While MRI is highly accurate for diagnosing DIE, the interpretation of MRI results hinges on the radiologist's proficiency in imaging techniques and comprehension of specific MRI findings [8]. The challenges in interpreting imaging findings for endometriosis are further compounded by the subtle manifestations of the disease, which can be overlooked or mistaken for other conditions. Consequently, patients often encounter delayed diagnosis, leading to misdiagnosis and adversely affecting their quality of life. Radiologists play a pivotal role in facilitating early and accurate diagnosis through MRI, providing detailed reports that assist in treatment planning and ultimately improve patient outcomes [8].

Reliability and Standardization of Biomarkers

The reliability and standardization of biomarkers for diagnosing endometriosis are critical factors that directly influence their effectiveness in clinical practice. Despite numerous studies aiming to identify non-invasive biomarkers for this condition, challenges persist due to variabilities in study design, a lack of consensus on the disease's pathophysiology, and the absence of specific symptoms, leading to delayed diagnosis. The gold standard for diagnosing endometriosis remains invasive surgery followed by histopathological examination, underscoring the urgent need for more reliable and standardized non-invasive biomarkers [12,13,34]. The research underscores the importance of developing biomarker panels rather than relying on single biomolecules for diagnosing endometriosis. While various biomolecules hold promise, they must demonstrate the requisite sensitivity and specificity for accurate diagnosis. Utilizing multiple biomarkers or a combination of different non-invasive diagnostic methods will likely enhance the reliability of diagnosing endometriosis. Future advancements in omics technology and immunoassay techniques offer the potential for discovering more valuable biomarker panels, emphasizing the necessity for standardized and reliable biomarkers to diagnose endometriosis [13].

Socioeconomic and Cultural Barriers to Accessing Diagnosis

Socioeconomic and cultural barriers exert a significant influence on accessing timely diagnosis for endometriosis. Research underscores disparities in access to care, diagnosis, treatment, and management of endometriosis among various racial and socioeconomic groups in the United States. Studies reveal that non-White women encounter challenges in receiving appropriate care, with Black women experiencing elevated rates of perioperative complications, mortality, and prolonged perioperative stages compared to other racial and ethnic groups. These disparities underscore the imperative for further research to address diagnostic and treatment gaps beyond surgical management and socioeconomic hurdles [35,36]. Cultural factors also have a crucial impact on delayed diagnosis, as societal perceptions of womanhood and menstruation may normalize symptoms, impeding healthcare providers' recognition of endometriosis. Furthermore, the stigma surrounding menstruation and the misconception that endometriosis primarily affects white, middle-class women contribute to diagnostic biases and inadequate care for individuals from diverse racial and ethnic backgrounds. These cultural beliefs, coupled with socioeconomic inequities, erect substantial barriers to accessing proper diagnosis and treatment for endometriosis, underscoring the necessity of addressing these issues to enhance healthcare equity and outcomes for all individuals affected by the condition [37].

Future directions in endometriosis diagnosis

Advances in Non-invasive Diagnostic Techniques

Advancements in non-invasive diagnostic techniques for various skin disorders, including psoriasis and pigmentary skin disorders, have enhanced diagnostic accuracy and patient comfort. These innovations encompass a spectrum of imaging techniques such as dermoscopy, high-frequency ultrasound, multispectral imaging, optical coherence tomography, reflectance confocal microscopy, and more. By offering detailed insights into skin properties in vivo, these methods facilitate the definitive diagnosis and therapeutic monitoring of conditions like psoriasis [38,39]. Moreover, non-invasive diagnostic modalities like dermoscopy, ultrasonography, confocal laser microscopy, and reflectance spectrophotometers have yielded promising results in diagnosing pigmentary skin disorders and cutaneous cancers. These techniques provide a comfortable and objective means to monitor disease progression and deliver accurate diagnoses without necessitating invasive procedures such as skin biopsies [38].

Integration of Artificial Intelligence in Diagnostic Algorithms

Integrating artificial intelligence (AI) into diagnostic algorithms revolutionizes healthcare by significantly enhancing diagnostic precision, streamlining administrative tasks, and personalizing treatment plans. AI algorithms, particularly machine learning methods like deep learning, are employed to analyze medical data and images from various modalities such as X-rays, MRIs, CT scans, and ultrasound. These algorithms excel at identifying patterns and abnormalities that may be challenging for human practitioners to detect, resulting in more accurate and efficient diagnoses [40-43]. In medical imaging analysis, AI has successfully detected diseases, such as breast cancer, and identified lung nodules. It offers superior pattern recognition capabilities, consistency, speed, and the ability to rapidly process vast volumes of data. Additionally, AI is making strides in pathology by automating workflow processes in pathology labs and enhancing accuracy in tissue sample analysis and cancer diagnoses. Powered by AI, predictive diagnostics utilize patient data to forecast health risks and personalize risk assessments for diseases like diabetes and heart attacks [42]. Moreover, AI's impact extends to treatment planning through personalized medicine, accelerated drug development, and robot-assisted surgery. In personalized medicine, AI tailors treatment plans based on genetic profiles, lifestyle factors, and health conditions. It interprets genetic data to predict disease predispositions and responses to treatments. In drug development, AI expedites the discovery process by efficiently analyzing biological and chemical data, predicting drug interactions, reducing development costs, improving clinical trials, and identifying new applications for existing drugs [42].

Personalized Medicine Approaches

Personalized medicine approaches in endometriosis entail tailoring treatment based on individual patient preferences and genetic characteristics to enhance patient outcomes. These approaches consider factors such as patient preferences for treatment attributes and genetic variations contributing to the disease. For instance, a study implemented a personalized medicine approach to determine individualized drug doses for endometriosis patients, taking into account patient preferences for the safety and efficacy attributes of the medication. This involved simulating weighted attributes representing various patient profiles and adjusting drug dosages accordingly [44]. Additionally, PrecisionLife has acquired the Oxford Endometriosis Gene (OXEGENE) dataset from the University of Oxford to develop personalized treatments for endometriosis patients. PrecisionLife aims to identify genetic disparities among individuals with endometriosis and elucidate the underlying mechanisms driving the disease by analyzing genetic data obtained from surgically confirmed patients. This initiative endeavors to expedite reaching a personalized diagnosis and develop novel treatments by correlating biomarkers with patient-specific genetic profiles [45].

Importance of Multidisciplinary Collaboration

Multidisciplinary collaboration is pivotal in managing endometriosis, particularly in intricate cases like DIE. A multidisciplinary team approach involves specialists such as endometriosis surgeons, colorectal surgeons, urologists, radiologists, pain specialists, and psychologists collaborating to deliver optimal patient care. This collaborative approach ensures higher-quality decision-making, standardized patient care, and improved outcomes. Research indicates that a multidisciplinary approach enhances pain management, improves quality of life post-treatment, and improves patient outcomes in severe endometriosis cases [46,47]. The advantages of multidisciplinary collaboration extend beyond individual expertise by promoting cross-discipline learning, research, and review. This approach is crucial for addressing the complexity of endometriosis management and ensuring that patients receive comprehensive and evidence-based care. By bringing together specialists from diverse fields, a multidisciplinary team can devise personalized treatment plans, enhance postoperative outcomes, and provide holistic support to patients navigating endometriosis's physical, mental, and emotional challenges [46,47].

## Conclusions

This review has provided a comprehensive assessment of the diagnostic landscape of endometriosis, highlighting the strengths and limitations of current approaches. From clinical evaluation to advanced imaging and biomarker research, each method offers valuable insights into diagnosing this debilitating condition. Despite advancements, challenges such as delayed diagnosis and variability in diagnostic accuracy persist, necessitating collective action to improve detection and management. This requires increased awareness among healthcare providers and the public, investment in research for novel diagnostic modalities, and the implementation of standardized protocols in clinical practice. By fostering collaboration across disciplines and advocating for improved diagnostic strategies, we can enhance early detection, streamline patient care, and ultimately improve outcomes for individuals affected by endometriosis.
